# Structural characterization of membrane-bound human immunodeficiency virus-1 Gag matrix with neutron reflectometry

**DOI:** 10.1116/1.4983155

**Published:** 2017-05-16

**Authors:** Rebecca Eells, Marilia Barros, Kerry M. Scott, Ioannis Karageorgos, Frank Heinrich, Mathias Lösche

**Affiliations:** Department of Physics, Carnegie Mellon University, Pittsburgh, Pennsylvania 15213; Memorial Sloan Kettering Cancer Center, New York, New York 10065; Biomolecular Measurement Division, National Institute of Standards and Technology, Gaithersburg, Maryland 20899 and Institute for Bioscience and Biotechnology Research, Rockville, Maryland 20850; Department of Physics, Carnegie Mellon University, Pittsburgh, Pennsylvania 15213 and NIST Center for Neutron Research, National Institute of Standards and Technology, Gaithersburg, Maryland 20899; Departments of Physics and Biomedical Engineering, Carnegie Mellon University, Pittsburgh, Pennsylvania 15213 and NIST Center for Neutron Research, National Institute of Standards and Technology, Gaithersburg, Maryland 20899

## Abstract

The structural characterization of peripheral membrane proteins represents a tremendous challenge in structural biology due to their transient interaction with the membrane and the potential multitude of protein conformations during this interaction. Neutron reflectometry is uniquely suited to address this problem because of its ability to structurally characterize biological model systems nondestructively and under biomimetic conditions that retain full protein functionality. Being sensitive to only the membrane-bound fraction of a water-soluble peripheral protein, neutron reflectometry obtains a low-resolution average structure of the protein-membrane complex that is further refined using integrative modeling strategies. Here, the authors review the current technological state of biological neutron reflectometry exemplified by a detailed report on the structure determination of the myristoylated human immunodeficiency virus-1 (HIV-1) Gag matrix associated with phosphoserine-containing model membranes. The authors found that the HIV-1 Gag matrix is able to adopt different configurations at the membrane in a *p*H-dependent manner and that the myristate group orients the protein in a way that is conducive to PIP_2_-binding.

## I. INTRODUCTION

### A. Structural biology of peripheral membrane proteins

Water-soluble, peripheral membrane proteins perform a wide range of functions within the cell and are involved in cell signaling: the transfer of chemical information into and out of the cell, enzymatic activities on membrane components, regulation of integral membrane proteins, transport of small molecules or electrons, and structural support for the localization of proteins and protein complexes on the membrane. To achieve their broad range of functionalities, peripheral proteins, in many cases, interact only temporarily with lipid membranes or with receptor sites on integral membrane proteins.^1,2^ Even when interactions between the proteins and the membrane are transient, these interactions can lead to structural rearrangements or conformational changes in the proteins that allow them to perform their function.^3^ As such, structures of the solution state of these proteins often provide insufficient information to fully understand the biological processes in which they are involved. Reflectometry techniques, in particular neutron reflectometry (NR), offer distinct advantages over traditional structure determination methods, such as crystallography or NMR, for characterizing the protein in a biomimetic membrane environment even though at a lower intrinsic resolution.^4–7^ The development of in-plane fluid tethered lipid membranes for NR (Refs. 8–10) and the implementation of molecular modeling strategies^11^ have transformed the technique to allow for routine studies of membrane-associated proteins.^7,12^ NR experiments can be performed over a wide range of environmental conditions in terms of ionic strength, *p*H value, and temperature including—but not limited to—physiologically relevant ranges. NR can also characterize samples in a changing environment such that biological processes can be simulated *in situ*, for example by introducing cofactors or applying external cues between distinct measurements.^13^ A fully atomistic data interpretation can be achieved using integrated modeling strategies involving complementary experimental data and molecular dynamics (MD) simulations.^14–16^

### B. HIV-1 Gag MA

Here, we study the myristoylated membrane-targeting domain of human immunodeficiency virus-1 (HIV-1) Gag polyprotein. Gag is the structural factor essential for capsid formation of nascent daughter virus of HIV-1 and other retroviral species.^17^ Expressed in the infected host cell, capsid formation of the daughter virus requires Gag trafficking and binding to the plasma membrane (PM), interaction with neighboring Gag proteins to form the immature protein shell of the capsid, and the binding of viral RNA cosynthesized in the hijacked cell. From its N-terminus to its C-terminus, Gag contains four major structural domains to perform these functions: matrix (MA), capsid (CA), nucleocapsid (NC), and p6, as well as connecting spacer peptides.^18^ Membrane targeting of Gag is mediated by the myristoylated N-terminal MA domain. In solution, nonmyristoylated MA adopts a compact, globular fold formed by five α-helices near the N-terminus while the C-terminus is more flexible.^19–22^ Myristoylation does not result in large conformational rearrangements of this structure even though the protein is able to adopt two states in which the myristate is either sequestered or exposed to the cytosol.^23^

MA recognizes specific PM components and binds to the membrane surface by a variety of physical interactions. The myristate serves as a hydrophobic anchor,^24–26^ while a conserved patch of basic residues interacts electrostatically with negatively charged lipids in the inner leaflet of the PM.^25,27,28^ In addition, the protein exhibits specificity for phosphoinositol-4,5-diphosphate [PI(4,5)P_2_],^29–31^ a characteristic marker of the inner PM.^32^ The fully assembled Gag lattice that forms the protein backbone of the immature viral capsid with its lipid membrane acquired from the host PM has been studied by electron tomography.^33^ However, a dissection of the molecular mechanisms that attract the protein to the lipid surface requires a comparative study of the smaller MA domain under well-controlled synthetic conditions. We previously used NR to study the nonmyristoylated matrix and produced a structural model for the electrostatically driven membrane association.^34^ The protein was observed in a favorable orientation for Gag lattice formation, but it remained unclear if myristoylation would further modulate the structural organization of the protein at the membrane interface. Recently, we quantified the thermodynamics of MA binding to membranes of varying compositions using surface plasmon resonance (SPR) and studied the contribution of the myristate to membrane interactions.^35^ While SPR provided the energetics of membrane binding, it does not reveal structural information.

Here, we review recent improvements in neutron reflectometry and the required sample preparation. We then discuss the application of NR to HIV-1 myrMA with a particular focus on the challenges associated with studying a protein with multiple binding motifs and reduced solubility due to the myristoyl moiety. Finally, we report the structural organization of myrMA on charged membranes and compare it with (−myr)MA. Our results confirm the conformational flexibility of myrMA and show the effect of *p*H on the dynamics of the myristoyl sequestration pocket. The combination of hydrophobic and electrostatic interactions leads to a protein orientation, distinct from that under purely electrostatic association, in which key residues are favorably positioned for PIP_2_ binding.

## II. BIOLOGICAL NEUTRON REFLECTOMETRY

In a typical application to structural biology, NR provides a one-dimensional compositional profile along the normal vector of a lipid bilayer membrane. Thus, a component volume occupancy (CVO) profile along this direction is obtained that accounts for all molecular components of the interfacial architecture including the lipid bilayer and membrane-associated proteins, peptides, and small molecules. At every position along the bilayer normal, the CVO profile represents an in-plane average over planes that are parallel to the lipid bilayer. Thereby, NR yields a temporal and spatial ensemble average over, for example, membrane-associated protein configurations.

A competitive advantage of NR with respect to traditional techniques in structural biology is that the experiment can be carried out using a fully buffer-immersed sample and by maintaining a high flexibility regarding buffer conditions, such as ionic strength and *p*H. NR is nondestructive with the implication that the sample can be manipulated during a NR measurement series by changing, for example, the environmental conditions or adding other molecular cofactors. Biological processes can thus be mimicked, and the structural evolution of the system under study can be monitored.^13^ Specific isotopic labeling of a subset of proteins that form protein–protein complexes allows for the structural characterization of individual proteins within such a complex.^36,37^ In addition, NR provides particular advantages for the study of disordered proteins and peptides, as well as transiently bound peripheral proteins.^38,39^

NR is intrinsically a low-resolution technique. With current instrumentation, structural features with a thickness of at least ≈10 Å, for example, membrane-bound proteins, can be resolved with a spatial resolution as low as 1 Å. Volume occupancy profiles of components filling only 5%–10% of the volume at any position along the surface normal can be reliably determined using the methods outlined below.

### A. Biomimetic model membrane systems

A membrane model system useful for biological NR has to meet several criteria. As a technical requirement, it needs to be planar, of low interfacial roughness, long-time stable, and homogenous over a large sample area (cm^2^). High interfacial roughness and a curved interface negatively affect the resolution of the measurement.^40^ Long-time stability is required due to the comparatively low flux at current neutron sources and, therefore, long measurement times in NR amounting to several hours per condition. For the same reason, large sample sizes are advantageous as they make better use of the neutron beam. The homogeneity of the sample is required primarily to ensure a unique analysis of the NR data. Inhomogeneous bilayers, even on length scales below the coherence length of the neutron beam, require a more complex modeling and therefore lower the certainty with which the structural features of interest can be determined. For example, a high density of defects in the lipid bilayer constitutes a significant disadvantage for structural characterization of membrane-associated proteins.

As a biological requirement, a model membrane has to be representative of a lipid membrane *in vivo* and is ideally accessible to buffer and protein exchange during the measurement. A flexible model membrane system supports a wide range of relevant lipid compositions while maintaining lipid diffusion rates that are comparable to biological membranes. It is also structurally inert toward changes in environmental conditions—temperature, *p*H, or ionic strength—which helps to retain a focus on the structural changes induced by membrane-associated proteins.

We devised a family of sparsely-tethered lipid membranes (stBLMs) optimized for biological NR that fulfill these criteria.^8–10^ The tether molecules have a central polyethylene oxide chain of 6–9 repeated units, which are functionalized on one end with two hydrocarbon chains—either saturated or unsaturated—that integrate into the hydrocarbon core of a lipid bilayer membrane. The other end is functionalized with either a thiol or thiol acetate group that binds the tethered membrane to a gold-surface. The polyethylene oxide provides about 15 Å of hydrated space between the lipid bilayer and the gold support. Small thiolated molecules coadsorbed with the tethers compete for binding to the gold-support, thus lowering the density of membrane anchors and passivating exposed areas of the gold surface. The relatively thin submembrane space limits the types of proteins that can be studied to those that do not have large extra-membraneous domains on both sides of the lipid bilayer. On the other hand, it keeps the membrane interface flat and of low roughness, which benefits the resolution of the measurements. We demonstrated that diffusion rates in stBLMs are comparable to those observed in unilamellar vesicles^41^ and have not yet encountered any limitations in mimicking relevant *in vivo* lipid compositions with the stBLM system.

We integrated the stBLM platform into NR and complementary surface-sensitive characterization techniques such as SPR (Ref. 35) and electrochemical impedance spectroscopy.^14,42^ For peripheral membrane proteins, such complementary characterization is an indispensable tool to identify optimal conditions for structure determination.

### B. Composition-space modeling of neutron reflectometry

**Fig. 1.**
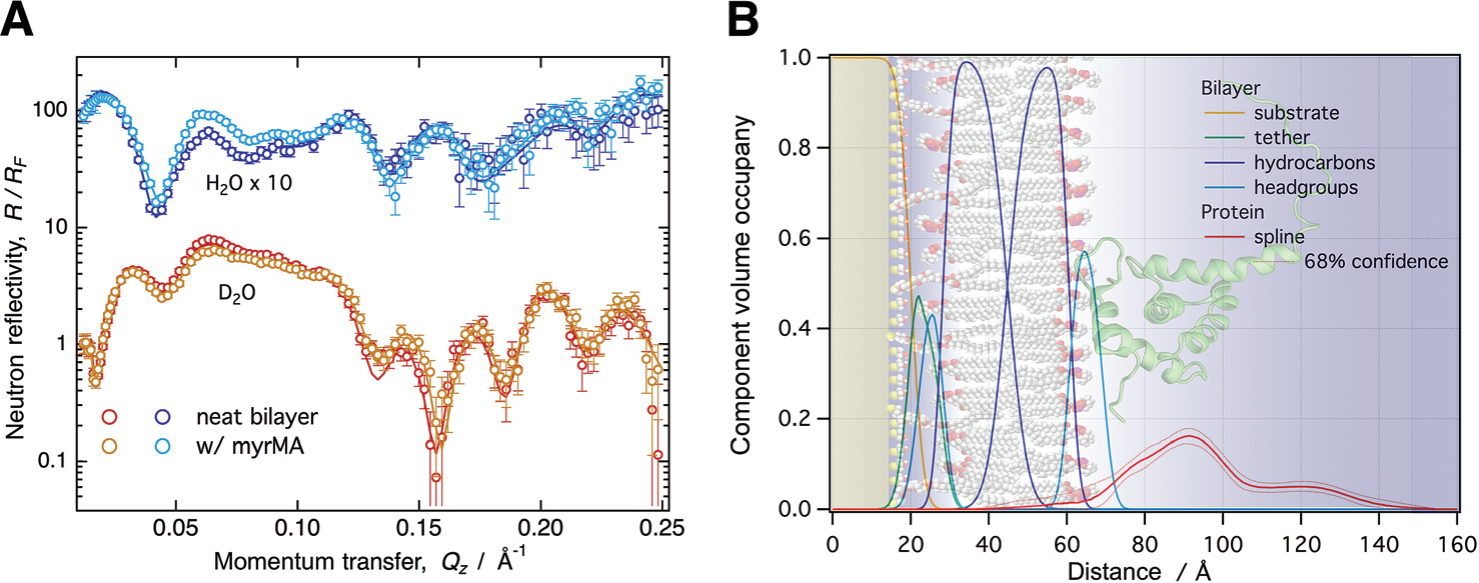
(a) NR curves normalized by the Fresnel reflectivity (*R_F_*) for a 50:50 DOPC:DOPS stBLM before and after protein addition (10 *μ*M myrMA, *p*H 8.0, 50 mM NaCl). Each condition was characterized using two isotopically distinct bulk solvents (H_2_O and D_2_O-based buffer) using *in situ* buffer exchange. (b) CVO profiles for the bilayer structure and the membrane-associated protein were obtained by composition-space modeling. The protein orientation was determined by rigid body modeling using the NMR structure in PDB entry 2H3F. The background image visualizes the resulting protein orientation on the stBLM surface.

Applications of NR in structural biology rely on modeling to yield structural information of membrane-bound proteins. This is best achieved using a molecular-scale description paired with an accurate determination of confidence limits on model parameters. We have consequently retired the traditional slab model used in NR,^43^ replacing it with a composition-space model that yields CVO profiles of molecular and submolecular groups (see Fig. 1).^7,11^ This model makes full use of auxiliary information such as molecular volumes and chemical connectivity, thereby reducing the number of fit parameters and increasing the confidence on unknown parts of the structure. In the case of a tethered lipid bilayer membrane, the volume occupancies of the headgroups are tied to the respective hydrocarbon chains, as individual volumes for both these parts of a lipid molecule are usually known from auxiliary methods such as x-ray diffraction.^44^ Therefore, aside from parameters for the tether molecules, the only three parameters that are required to describe all submolecular groups of a lipid bilayer are the thicknesses of the substrate-proximal and substrate-distal hydrocarbon chains and the bilayer completeness. Headgroup thicknesses are typically too small to be reliably determined by NR, and fixed values obtained, for example, from MD simulations are used instead.

In distinction to slab models, the composition-space approach allows for spatially overlapping molecular distributions. Molecular profiles of unknown shape, such as those of membrane-associated proteins, require free-form models. We implemented approaches based upon Hermite splines that accurately join protein profiles with CVO profiles of the molecular constituents of the lipid bilayer.^7^ An unbiased determination of modeling uncertainties—a necessity for free-form modeling due to the inherent risk of over-parameterization—is carried out using a Monte Carlo Markov Chain optimizer.^45^

### C. Integrative modeling of interfacial structures

The intrinsically low resolution of NR in 1D calls for integrative modeling strategies to obtain high-resolution 3D structures of membrane-bound proteins. We routinely integrate available crystallographic or NMR structures into the refinement of NR data from surface-associated proteins (see Fig. 1).^7,14,15^ A comparison of a free-form protein profile and profiles obtained using an atomistically resolved protein structure reveals whether the protein undergoes reorganization upon binding to the membrane. When varying the orientation of the high-resolution protein structure with respect to the membrane (rigid body modeling), such a comparison can determine its orientation if the protein does not undergo significant structural changes, within the resolution of NR, upon binding. However, if no agreement with the free-form profile can be achieved, significant structural changes are evident that require additional modeling. Such an alternative strategy generally involves MD or Monte Carlo simulations of the membrane-bound protein, which can be conducted with steering by the experimental data. Additional experimental data, for example local structural information from NMR, can also be integrated in the form of constraining potentials in the simulation. While we have started utilizing simulation-based integrative modeling strategies,^15,16,38,46^ a complete integrative modeling framework for NR has not yet been established.

## III. APPLICATION: THE MEMBRANE BOUND STRUCTURE OF THE MYRISTOYLATED HIV-1 GAG MATRIX

### A. Materials and methods

#### 1. Protein expression and purification

Standard laboratory chemicals, culture media, myristic acid, isopropyl-β-D-thio-galactopyranoside (IPTG), and phenylmethylsulphonylfluoride (PMSF) were purchased from Sigma-Aldrich (St. Louis, MO), unless otherwise noted. Tris(2-carboxyethyl)phosphine hydrochloride (TCEP-HCl) was purchased from Thermo Fisher Scientific (Waltham, MA). The plasmid used for the myrMA preparation via coexpression of MA protein and *N*-myristoyltransferase was kindly provided by Michael Summers (University of Maryland Baltimore County). Transformed *Escherichia coli* BL21 (DE3) cells containing the expression vector were grown while shaking (250 Hz) at 37 °C to OD_600_ = 0.4. Cells were supplemented with 1 ml of myristic acid (10 mg/ml) per liter of culture and kept growing to OD_600_ = 0.8. At this point, protein expression was induced by adding IPTG to a concentration of 1 mmol/l (1 mM), and cells were kept at 30 °C overnight. The cells were harvested by centrifugation at 6000*g* for 15 min at 4 °C, washed with phosphate-buffered saline, and held frozen at −80 °C. Five grams (wet-weight) of cells were resuspended in 30 ml of lysis buffer [20 mM Tris, 300 mM NaCl, 10% glycerol, 1 mM PMSF, 1× protease inhibitor mixture set I (Calbiochem; EMD Millipore, Billerica, MA), and 1 mM TCEP, *p*H 7.4] and disrupted by sonication on ice. The cell lysate was centrifuged at 10 000*g* for 30 min at 4 °C, and the protein was purified by immobilized metal affinity chromatography. Monomeric MA was separated by size exclusion chromatography on a Superdex-75 10/30 GL column using an AKTA purifier system (Amersham Biosciences, Little Chalfont, UK).

#### 2. Mass spectrometry

Protein identification and purity were established using an electrospray ionization interface on an Agilent 6550 quadrupole time of flight mass spectrometer coupled with an Agilent 1200 high performance liquid chromatography column (Santa Clara, CA). Protein was eluted from a C18 column (3 *μ*m, 3 × 150 mm; Waters, Milford, MA) over a 30 min gradient from 3% to 60% acetonitrile containing 0.1% formic acid at a flow rate of 9 *μ*l/min. Data were acquired in the positive ion mode with the following settings: capillary temperature, 290 °C; capillary voltage, 3500 V; fragmentor, 300 V; and a *m/z* 300–3200 mass range. Mass deconvolution was performed using the Agilent MassHunter (version B.06) software.

Peptic peptides of myrMA were generated by passing 17 pmol of protein through an Enzymate pepsin column (Waters, Milford, MA) and identified using tandem mass spectrometry (MS/MS) on a Thermo LTQ Orbitrap Elite unit (Thermo Fisher Scientific, Waltham, MA). One full mass spectral acquisition triggered six scans of MS/MS with activation by collision-induced dissociation on the most abundant precursor ions. Peptides were identified using the MASCOT (Matrix Science, Oxford, UK) database search engine with the following parameters: enzyme, none; oxidation (M) as a variable modification; MS tolerance, 20 ppm; MS/MS tolerance, 0.6 Da; peptide charge of +2, +3, and +4.

#### 3. HDX-MS and HDX data processing

D_2_O was obtained from Cambridge Isotope Laboratories Inc. (Andover, MA). For hydrogen-deuterium exchange MS (HDX-MS) analyses, the myrMA protein stock was diluted in H_2_O buffer (20 mmol/l Tris, 150 mmol/l sodium chloride, 2 mmol/l TCEP at *p*H 7.4 and 8.0) to prepare a 5 *μ*mol/l final concentration, and equilibrated at 1 °C. HDX was conducted on a HDX PAL robot (LEAP Technologies, Carrboro, NC). Protein solutions (5 *μ*l) were diluted in 25 *μ*l of D_2_O buffer (20 mmol/l Tris, 150 mmol/l sodium chloride, 2 mmol/L TCEP at *p*H 7.4 and 8.0) at 25 °C. At the selected times (0 s, 30 s, 5 min, 15 min, 1 h, and 4 h), the HDX sample was quenched by mixing with 35 *μ*l of quench buffer (3 mol/l urea, 0.1 mol/l sodium phosphate at *p*H 2.5) at 1 °C. The quenched solution was injected into an on-line immobilized pepsin column for 3 min. The digested protein solution was trapped on a C18 guard column (1.0 mm diameter × 10 cm length, 5 *μ*m; Grace Discovery Sciences, Deerfield, IL) and separated with a C18 analytical column (1.0 mm diameter × 5 cm length, 1.9 *μ*m, Hypersil GOLD; Thermo Fisher Scientific, Waltham, MA) via a Dionex Ultimate 3000 UPLC with a 9.5 min gradient operated with a binary mixture of solvents, A (water containing 0.1% formic acid) and B (80% acetonitrile and 20% water containing 0.1% formic acid), at a flow rate of 50 *μ*l/min. The gradient settings were: 5%–35% solvent B for 3 min, 35%–60% solvent B for 5 min, 60%–100% solvent B for 0.5 min, isocratic flow at 100% solvent B for 0.5 min, and a return in 5% solvent B for 0.5 min. LC connection lines and valves were housed in a refrigerated compartment at 2 °C. Peptides were analyzed on a Thermo Orbitrap Elite unit (Thermo Fisher Scientific, Waltham, MA). The instrument settings were: spray voltage, 3.7 kV; sheath gas flow rate, 25 (arbitrary units); capillary temperature, 275 °C. In the Orbitrap stage, MS spectra were acquired with the resolution set at 60 000. Three replicates for each ion-exchange time point were obtained.

From the mass spectra obtained during HDX-MS experiments, the centroid of each deuterated peptide envelope and the relative deuterium uptake by each peptide were calculated using HDX WorkBench (Scripps Research Institute, Jupiter, FL). Corrections for back exchange were made by considering the values of 80% deuterium content of the exchange buffer and an estimated 70% deuterium recovery. Paired t-tests were used to verify deuterium uptake differences.

#### 4. Lipid and liposome preparation

1,2-dioleoyl-*sn-*glycero-3-phosphocholine (DOPC), 1,2-dioleoyl-*sn-*glycero-3-phospho-L-serine (DOPS), 1-palmitoyl-2-oleoyl-*sn*-glycero-3-phosphocholine (POPC), 1-palmitoyl-d_31_–2-oleoyl-*sn*-glycero-3-phosphocholine (d_31_-POPC), and 1-palmitoyl-2-oleoyl-*sn*-glycero-3-phospho-L-serine (POPS) were all purchased from Avanti Polar Lipids, Inc. The tether compound HC18 [Z20–(Z-octadec-9-enyloxy)-3,6,9,12,15,18,22-heptaoxatetracont-31-ene-1-thiolacetate] was synthesized and characterized as described.^10^ Lipids from stock solutions in chloroform were mixed at desired molar ratios. The organic solvent was removed from the lipid mixtures via evaporation under vacuum for 12 h. The lipid films were hydrated in high salt aqueous buffer (1 M NaCl, 10 mM NaPO_4_ at *p*H 7.4) to a lipid concentration of 5 mg/ml and then sonicated until clear.

#### 5. Preparation of sparsely tethered bilayer lipid membranes

1 × 3 in. microscopy glass slides (Thermo Fisher Scientific, Waltham, MA; for SPR) and 3 in. diameter, 5 mm thick n-type Si:P[100] wafers (El-Cat Inc., Ridgefield Park, NJ; for NR) were cleaned with 5 vol. % Hellmanex solution (Hellma Analytics, Müllheim, Germany) and then sulphuric acid plus Nochromix (Godax Laboratories, Cabin John, MD) followed by extensive rinsing with ultrapure water (EMD Millipore, Billerica, MA) and pure ethanol (EtOH, Pharmo-Aaper, Shelbyville, KY) and dried in a N_2_ gas stream. The substrates were coated with Cr (∼20 Å) and Au (∼150 and ∼450 Å for NR and SPR, respectively) by magnetron sputtering (ATC Orion; AJA International, Scituate, MA). Coated substrates were immediately soaked in a 7:3 (mol/mol) ethanol solution of HC18 and ß-mercaptoethanol (ßME) at a total concentration of 0.2 mM to form a self-assembled monolayer (SAM). A 5:5 (mol/mol) HC18:ßME ratio was used for the preparation of the 70:30 d_31_-POPC:POPS stBLM for NR to aid complete bilayer formation. Vesicle solutions were allowed to incubate the dry SAM for ∼2 h, and then the system was flushed with low ionic strength buffer (50 mM NaCl, 10 mM NaPO_4_, *p*H 7.4) to complete stBLM formation.

#### 6. Neutron reflectometry

NR measurements were performed at the NG7 horizontal and CGD-Magik^47^ reflectometers at the NIST Center for Neutron Research (NCNR). Reflectivity curves were recorded for momentum transfer values 0.01 ≤ *q*_z_ ≤ 0.25 Å^−1^. For each measurement, adequate counting statistics were obtained after 5–7 h. The NCNR flow cell^45^ allows for *in situ* buffer exchange; thereby, subsequent measurements were performed on the same sample area. The entire flow cell was maintained at room temperature (RT). After *in situ* completion of the stBLM, NR data were sequentially collected with H_2_O buffer and D_2_O buffer in the measurement cell. Buffer exchange was accomplished by flushing ∼10 ml of buffer through the cell (volume ∼ 1.3 ml) using a syringe.

Two methods were used to prepare protein for the measurement. If the protein stock buffer did not match the desired working buffer, spin columns (Micro Bio-Spin 6 Columns; Bio-Rad, Hercules, CA) were used for buffer exchange followed by a measurement of the protein concentration post exchange. Protein at the desired concentration (typically 10 *μ*M) was then prepared by diluting an aliquot from the stock with the working buffer (H_2_O or D_2_O) to a final volume of ∼1.5 ml. If the protein stock buffer was the same as the working buffer, an aliquot of protein from the main stock was diluted to achieve the desired concentration. Protein was introduced to the NR cell via a syringe, and its incubation with the bilayer was measured in both H_2_O and D_2_O buffer contrasts. The system was then rinsed and measured in both contrasts again to detect any remaining, tightly bound protein at the membrane interface.

1D-structural profiles along the lipid bilayer normal were parameterized using a stratified slab model for the solid substrate,^43^ a continuous distribution model for the stBLM,^11^ and a monotonic Hermite spline for the model-free protein distribution.^7^ Individual slabs were implemented for the bulk silicon, silicon oxide, chromium, and gold layers. Fit parameters are thickness and neutron scattering length density (nSLD) for each layer, except for the bulk silicon for which the nSLD is known. One global roughness fit parameter was applied to all the substrate interfaces. Individual submolecular groups implemented in the continuous distribution model were: βME, tether polyethylene glycol chains, tether glycerol groups, substrate-proximal and substrate-distal phosphatidylcholine and phosphatidylserine (PS) headgroups, substrate-proximal and substrate-distal methylene chains and methyls of lipid and tether molecules. Fit parameters were the bilayer hydrocarbon thickness for each bilayer leaflet, bilayer completeness, tether surface density, tether thickness, and βME surface density. One roughness fit parameter was applied to all distributions.

The Hermite spline that described the CVO profiles of the protein was defined by control points which were on average 15 Å apart. The spatial extension of the protein along the bilayer normal determined the number of control points which were iteratively refined during model optimization. Fit parameters for each control point were the volume occupancy of the envelope and deviations from the equidistant separation of control points.

To determine the orientation of myrMA on the membrane, a high-resolution NMR structure [protein data bank (PDB) entry 2H3F]^30^ was used within a rigid body modeling approach after hydrogens were added using MolProbity.^48^ While this structure describes the unmyristoylated form of the protein, it is identical within the NR resolution to the NMR structure for myrMA (PDB entry 1UPH^23^) for the compact core-region of MA, residues Val-7–Ile-104 (0.5 ± 0.1 Å root mean square deviation).^23^ The conformation of the N-terminus depends on the state of the myristoyl group (sequestered, free, membrane-inserted), and we expect that the structure of the N-terminal in PDB entry 2H3F would best describe that of the membrane-bound state.

Two continuous fit parameters, the two Euler angles β and γ, describe the protein orientation at the interface. The reference orientation (β, γ) = (0°, 0°) is defined by the orientation of the protein given in PDB entry 2H3F and in the reference orientation; by definition, the intrinsic protein coordinate system and the extrinsic bilayer coordinate system are aligned. The *z*-axis in the PDB file is aligned parallel to the bilayer normal and points toward the substrate-distal side of the membrane. The *x* and *y*-axes are parallel to the plane of the bilayer. Orientations (β, γ) are then obtained by extrinsic rotations of the protein around the axes of the bilayer coordinate system. First, the protein is rotated by 0° ≤ γ < 360° counter-clockwise about the membrane normal, *z*. Then, the protein is rotated by 0° ≤ β ≤ 90° about the *x*-axis of the bilayer coordinate system (extrinsic rotation) that generally differs from a rotation around the (intrinsic) protein coordinate system at this point.

Optimization of model parameters was performed using the ga_refl and Refl1D software packages developed at the NCNR.^45^ All the reflectivity curves of one dataset were fit simultaneously with the same model, sharing fit parameters, for example, for the solid substrate. A Monte Carlo Markov Chain-based global optimizer^45^ was used to determine fit parameter confidence limits.

#### 7. Surface plasmon resonance

SPR measurements were conducted in a single-batch mode at (25.00 ± 0.01)  °C using a custom-built instrument (SPR Biosystems, Germantown, MD). Gold-coated glass slides with a SAM layer were assembled in the Kretschmann configuration by index matching to a prism and stBLMs prepared via vesicle fusion *in situ*, as described above. A 2D-CCD detector records the intensity of light reflected from the glass/buffer interface that carries the membrane, and the position of the intensity minimum is recorded as a function of time. SPARia (SPR Biosystems) was used for real-time acquisition of the response, *R*, measured as the displacement of the reflection minimum on the CCD (in pixels). To determine a baseline, the neat bilayer was measured before adding protein in increasing concentrations. Time courses of *R* were recorded for each protein concentration, *c_p_*, until equilibrated at *R_eq_.* The change in *R_eq_* as a function of *c_p_* was fitted with a Langmuir isotherm,

$$R_{eq}=\frac{R_{\infty }\cdot c_{p}}{c_{p}+K_{d}},$$

where *K_d_* is the equilibrium dissociation constant and *R_∞_* is the saturation of the SPR response as c → ∞.

### B. Results and discussion

#### 1. MA membrane binding depends on *p*H

Biophysical studies of protein membrane binding are ideally carried out under physiological conditions to allow for an extrapolation of the experimental results to the *in vivo* situation. In practice, however, this is often impossible. As a truncation product of full-length Gag, the isolated MA domain studied here lacks self-interaction mechanisms that promote Gag multimerization at the membrane via CA dimerization and RNA-binding by NC.^49^ Therefore, a reduction in the ionic strength was necessary to shift the binding equilibrium of MA for a characterization of the membrane-bound state.^34,35^ Other techniques, such as NMR, required a lower *p*H to permit measurements of protein–lipid interactions.^31^

Based on a comprehensive set of myrMA binding data to lipid membranes,^35^ optimal buffer conditions for a structural characterization using NR were found to be *p*H 7.4 and 50 mM NaCl where the membrane dissociation constant is *K_d_* ∼ 5 *μ*M. While SPR indicated Langmuir binding behavior, NR revealed membrane remodeling and proteinaceous multilayers at the interface under these conditions, which prohibited a conclusive structural characterization of membrane-bound myrMA, in contrast to (−myr)MA which forms well-defined protein monolayers at the membrane.^34^ NMR studies of myrMA showed that the myristate group can adopt sequestered and exposed states with only minor conformational changes.^23^ MA oligomerization has been associated with myristate exposure and also depends on protein concentration and *p*H.^23,50^ At *p*H 7.0, the monomer/trimer dissociation constant is (1.4 ± 0.2) 10^−8^ M^2^, whereas at *p*H 8, myrMA was found to be purely monomeric.^50^ At a protein concentration of 10 *μ*M used in NR, the trimer concentration is <50 nM, insignificant for membrane binding. However, if the trimer assembles in a lattice-competent structure, binding interfaces from more than one monomer would lead to a significant enhancement in binding and could explain the formation of protein multilayers.

**Fig. 2.**
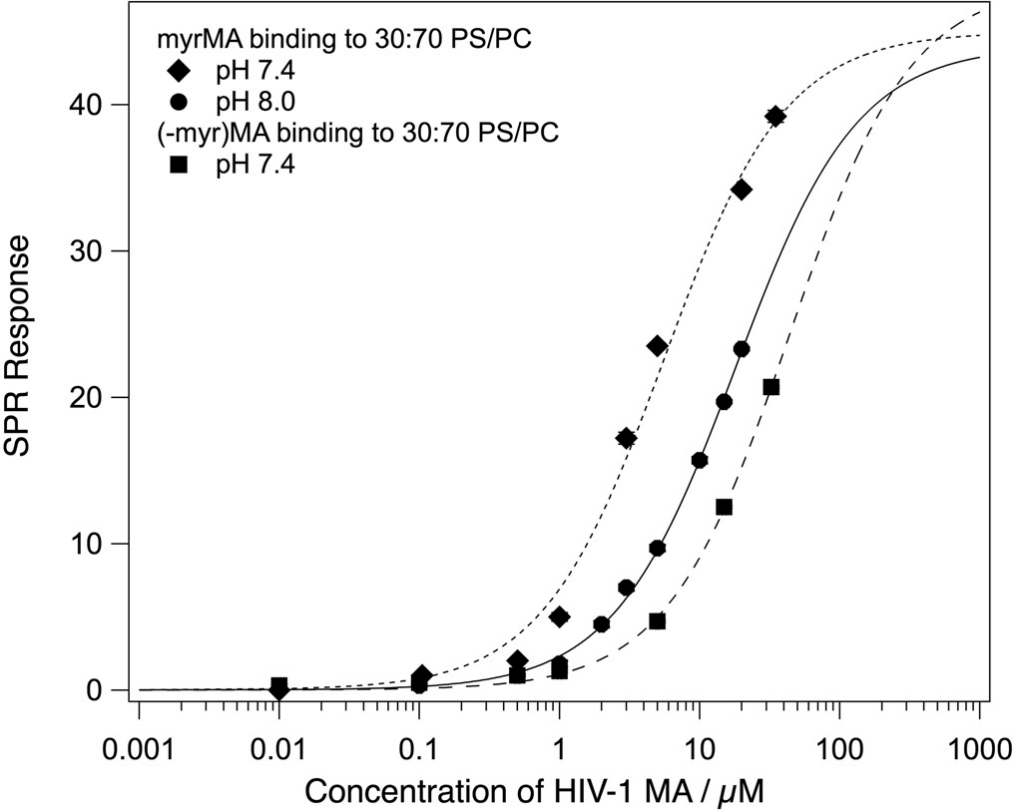
MA binding to stBLMs containing 30% PS at 50 mM NaCl. The equilibrium response is plotted as a function of MA monomer concentration and fitted with the Langmuir isotherm, and the binding curve for (−myr)MA at *p*H 7.4 is included for comparison. *K_d_* of myrMA increases from ∼5 to ∼18 *μ*M between *p*H 7.4 and 8 but remains significantly lower than for (−myr)MA at either *p*H. This shows that the myristate still contributes to membrane binding at *p*H 8.

An increase in the buffer *p*H to 8.0 shifted the monomer/trimer equilibrium entirely toward the monomer. SPR experiments on a 70:30 POPC:POPS stBLM showed a reduction in the binding affinity by a factor of ∼4 (Fig. 2), consistent with a shift toward the myristoyl-sequestered state^31,50^ and a change in protein charge from +3.7*e* to +2.9*e.*^51^ The saturation surface coverage was not significantly affected by the *p*H change, and the binding curve is well described by the Langmuir model.

#### 2. *p*H Dependence of MA conformational flexibility

**Fig. 3.**
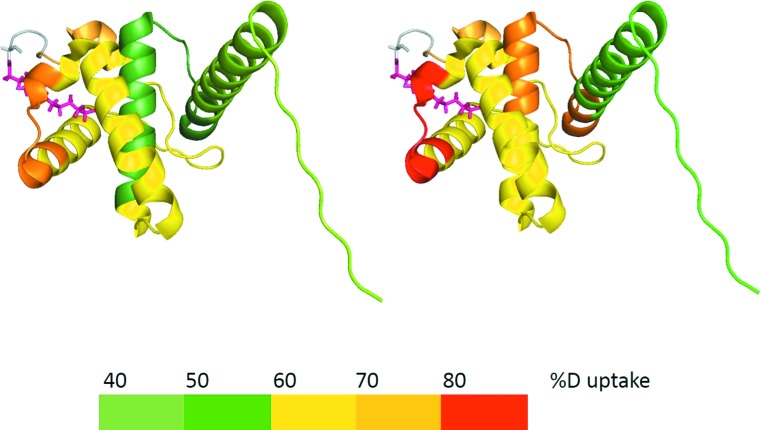
Deuteration profile of myrMA at *p*H 7.4 (left) and *p*H 8.0 (right) after 30 min of incubation in D_2_O.

myrMA was analyzed by HDX-MS to probe the effect of *p*H on the conformational flexibility in solution and to correlate dynamic changes with myristic acid exposure. The experiments detect the exchange of backbone amide hydrogen atoms of individual amino acids and reveal the extent of amide hydrogen bonding and solvent accessibility of a protein. By comparing measurements for different conditions, such as varying *p*H, changes in protein conformation and/or dynamics can be determined. The data (see supplementary material, Figs. S1–S4, for more detail)^55^ reveal that 34% of reporting amides were affected by *p*H changes from 7.4 to 8.0 and exhibit higher deuterium uptake rates over time between 6% and 30% (Fig. S5). The remaining 66% of the protein was not affected by *p*H changes. The overall deuterium uptake rate increase suggests that myrMA becomes more dynamic in a number of distinct regions at *p*H 8.0, and the majority of those regions belong to the helices that form the myristate pocket (see Fig. 3). We conclude that the higher *p*H increases the flexibility of the pocket, which promotes myristate sequestration at *p*H 8.0 in accordance with SPR binding data and literature.^23,50^

#### 3. MA adopts different configurations at the membrane depending on myristoylation, *p*H, and surface charge

**Fig. 4.**
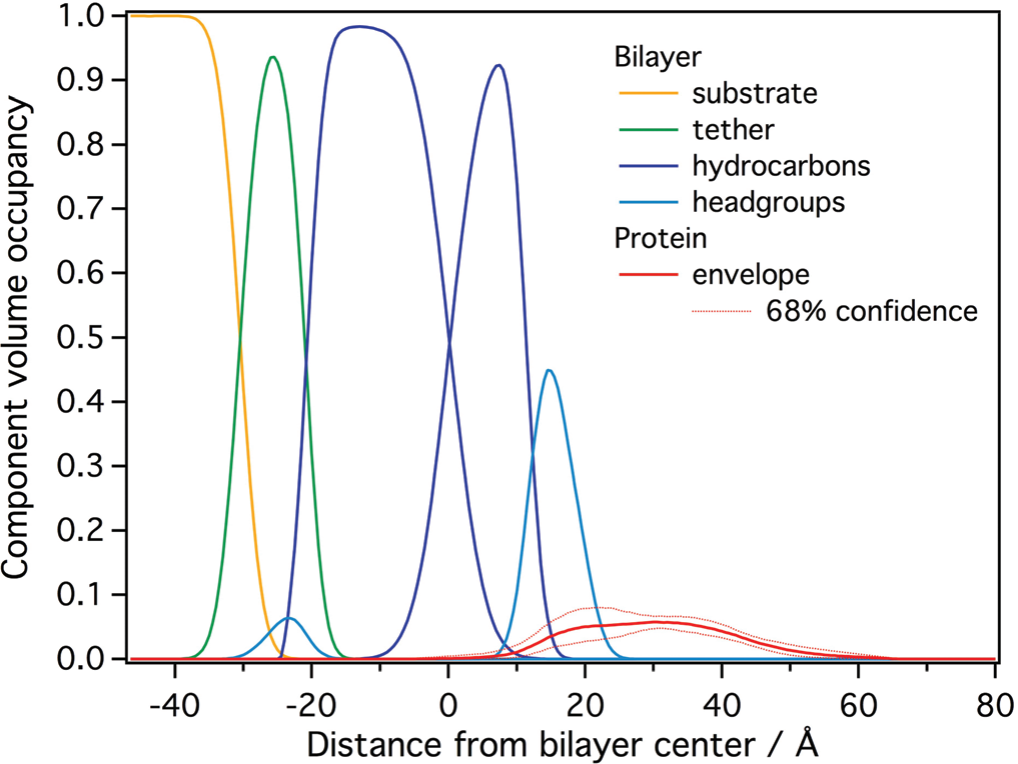
NR CVO profile of myrMA on a 70:30 d_31_-POPC:POPS stBLM at *p*H 8.0 after incubation with 10 *μ*M protein. The median protein envelope, shown with 68% confidence intervals (red traces), is inconsistent with a single MA conformation at the membrane interface.

To directly probe if the optimized conditions prevent the formation of overlayers, 10 *μ*M myrMA was studied with NR at a 70:30 d_31_-POPC:POPS stBLM at *p*H 8 and 50 mM NaCl (see Fig. S6). The thus obtained free-form protein CVO profile indicated a single layer of protein at the membrane interface (Fig. 4). However, a detailed analysis revealed that this profile is inconsistent with a single protein conformation at the membrane as it exhibits a broad maximum that exceeds the dimensions of a single MA molecule by ≈50%. Rinsing the sample with buffer did not significantly alter the profile (data not shown), indicating stable and irreversible protein binding. This result agrees with coarse-grained MD simulations of myrMA (Ref. 52) and electrostatic modeling^53^ that suggested dynamic binding of myrMA in multiple conformations to the membrane. We concluded that a slight tendency for protein multimerization at the interface remains a possibility. In addition, the low volume occupancy of the protein (5%–10%) leaves the analysis vulnerable to small systematic errors, which may be as large as 3%, in the CVO profile.

**Fig. 5.**
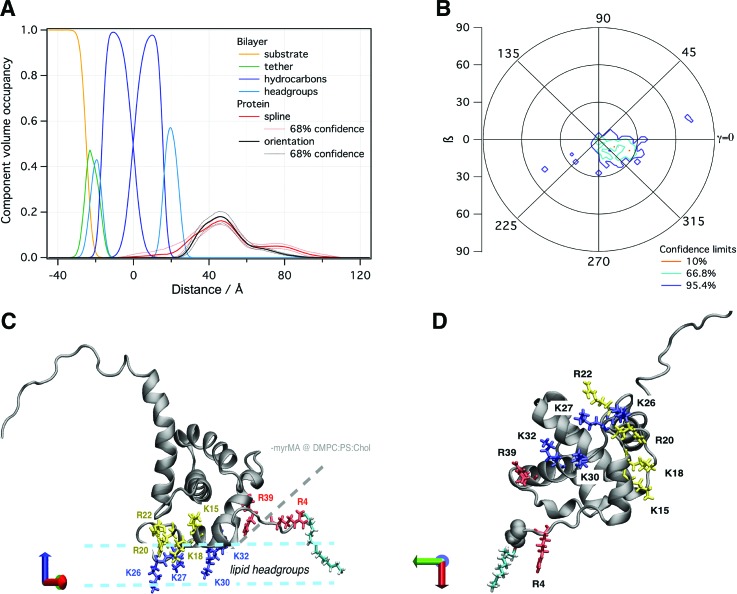
(a) NR CVO profile of myrMA on a 50:50 DOPS/DOPC stBLM at *p*H 8, 50 mM NaCl after incubation with 10 *μ*M protein. The median protein envelope is shown with 68% confidence intervals (red traces). The median orientation fit using the MA NMR structure (PDB entry 2H3F) is shown for comparison (black trace). (b) Probability distribution of myrMA orientations with respect to the 50:50 DOPS/DOPC bilayer normal. (c) The most likely orientation of the protein (β ∼ 20°, γ ∼ 335°) on the membrane. Lysine residues (K26, K27, K30, and K32) that penetrate deeply into the lipid headgroup region are highlighted in blue. Basic residues with a peripheral interaction (K15 and R22) or slight penetration (K18 and R20) are shown in yellow. Arginine residues (R4 and R39) that were previously shown in (−myr)MA to interact closely with the membrane but are more peripheral in myrMA are shown in red. A myristate group, shown in cyan, was added to the protein structure to highlight its location. (d) Bottom view of myrMA in its membrane bound orientation.

In a subsequent NR experiment, we increased the fraction of PS to 50% to yield a higher protein surface coverage and provide a more homogeneous binding interface, potentially reducing the propensity of the protein to adopt multiple configurations at the membrane [Fig. 1(a)]. These conditions, indeed, yielded a CVO profile compatible with a single myrMA configuration at the membrane [see Figs. 1(b) and 5(a)]. The orientation of myrMA was determined using the ensemble average of the NMR structures in PDB entry 2H3F [see Fig. 5(b)]. A comparison of the free-form CVO spline profile with the result of the rigid body modeling shows a very good agreement for the folded core but an imperfect agreement for the flexible N and C termini. The observed extra density of the spline CVO in the membrane region is consistent with myristate insertion and the insertion of N-terminal residues. This interpretation is supported by recent NR results that indicated a deep penetration of a peptide that represents myrMA truncated to its residues 2–32 into the hydrocarbon region of the lipid bilayer.^54^ The observed discrepancy between the free-form spline CVO profile and the profile based upon the NMR structure in the flexible C-terminal region shows that the ensemble of solution structures is too compact. While the orientation of the myrMA at the membrane is largely constrained by the asymmetric rigidly folded core, the discrepancies in both the termini introduce some uncertainty to the final result. Ultimately, an integrative modeling approach will be required to find a fully satisfying interpretation of the free-from profile of the membrane-associated myrMA.

In the most likely orientation of myrMA at the membrane [β ∼ 20°, γ ∼ 335°, shown in Fig. 5(b)], helix I and residues 31–35 of helix II are in close contact with the lipid membrane surface. While the overall membrane penetration is shallow, residues in the highly basic region [HBR; residues 15–31 (Ref. 25)] penetrate more deeply into the bilayer. In particular, lysine residues K26, K27, K30, and K32 penetrate deeply into the headgroup layer. Based on simulations and the previous NR measurements of (−myr)MA, these residues mediate membrane interactions via electrostatic interactions when PIP_2_ cannot be engaged.^34,52^ When PIP_2_ is present, these residues are still involved in membrane interactions. K30 and K32, specifically, were shown to be important for PIP_2_ binding,^31^ and mutation of these residues to glutamate retargeted Gag to intracellular compartments.^28^ In addition, R4 was also identified to interact with the membrane,^31,34^ but rigid body modeling of the NR data does not place this residue in direct contact with the headgroup region. On the other hand, with the proposed flexibility of the N terminus, R4 may penetrate the lipid headgroups as well.

Other HBR residues also interact closely with the membrane in our NR model: K15 and K18 from helix I and R20 and R22 from the loop connecting helix I and II. Based on the rigid body modeling, K18 and R20 penetrate slightly into the headgroup layer, and K15 and R22 may also insert into the headgroup region depending on their side-chain conformations. The NR characterization of (−myr)MA also identified these residues as interacting with the membrane, albeit more peripherally. Notably, R39, which showed a significant overlap with the headgroup region for (−myr)MA, is further away from the membrane surface for myrMA. The change in the orientation between the (−myr)MA and myrMA structures brings helix I and the HBR into more direct contact with the membrane surface while positioning R39 on helix II further away [Fig. 5(c)]. In simulations, a similar change in orientation was observed between the myristate exposed and myristate sequestered states, and myristate–membrane interactions resulted in a similar shift in membrane contacts from helix II to helix I.^52^ Although the membranes used in this study do not contain PIP_2_, our NR results show that the myristate interaction with the membrane positions the HBR such that it is conducive to PIP_2_ binding.

## IV. CONCLUSION

Structural characterization of peripheral membrane proteins poses unique challenges due to their interaction with the membrane, which is often transient, and the variety of protein conformations that can occur during this interaction. Detailed knowledge of membrane binding characteristics and aggregation behavior of a protein is required before a structural characterization can be pursued. A deviation from *in vivo* conditions, for example, by decreasing the ionic strength or altering the *p*H of the buffer, is often necessary to stabilize one particular conformation.

The current study of myrMA is an important step toward a full structural characterization of HIV-1 Gag membrane binding and viral assembly. We identified experimental conditions that overcome the challenges presented by this particular peripheral membrane protein. Our results for membrane-bound myrMA showed deviations of the membrane-bound protein structure from the high-resolution NMR structure in solution. By comparing the myrMA structure with the previously determined structure of (−myr)MA, we observed that their membrane complexes are similar to each other. However, the presence of the myristate results in a reorientation of MA that positions the HBR to allow for PIP_2_ engagement. This orientation agrees with simulations that suggested strong interactions between the membrane and helix I for the myristate-exposed form of the protein. In addition, the key basic residues K26, K30, and K32 were found to interact with PS when PIP_2_ lipids could not be engaged, and we observe penetration of these residues into the lipid headgroup region of PS-containing membranes in the absence of PIP_2_.
